# Supplementary material for: Dysregulation of T Follicular Helper and Regulatory Cells in IRF5-SLE Homozygous Risk Carriers and Systemic Lupus Erythematosus Patients
Source: Cells. 2025 Mar 19;14(6):454. doi: 10.3390/cells14060454 (PMC11941281; doi:10.3390/cells14060454)
Supplement: Supplementary file 1 [file cells-14-00454-s001.zip › cells-3505008-supplementary.pdf]

Supplementary Materials

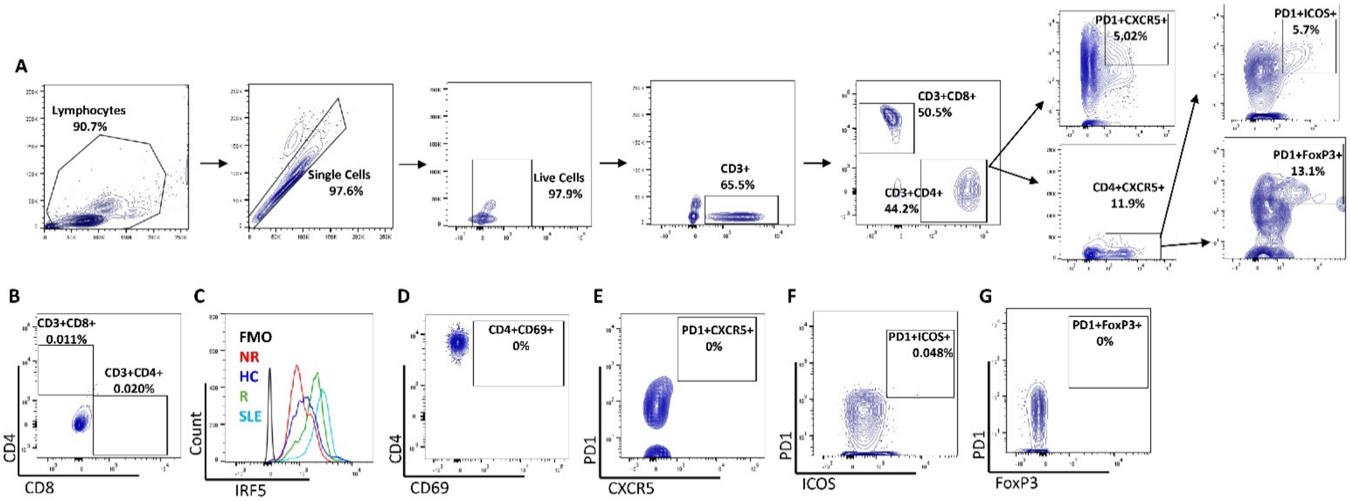

Supplemental Figure S1. Representative gating strategies and FMOs. **(A**) Representative gating strategy for circulating Tfh and Tfr cells. **(B)** FMO for CD4+ and CD8+ T cells. **(C)** Representative histogram plots showing IRF5 MFI within CD3+CD4+ T cells from a healthy risk (R) and non-risk (NR) carrier, non-genotyped healthy control (HC) and an SLE patient. **(D-G)** FMO for activated CD4+CD69+ cells **(D),** FMO for PD1+CXCR5+Tfh cells **(E),** FMO for PD1+ICOS+Tfh cells **(F)**, and FMO for PD1+FoxP3+Tfr cells **(G)**.

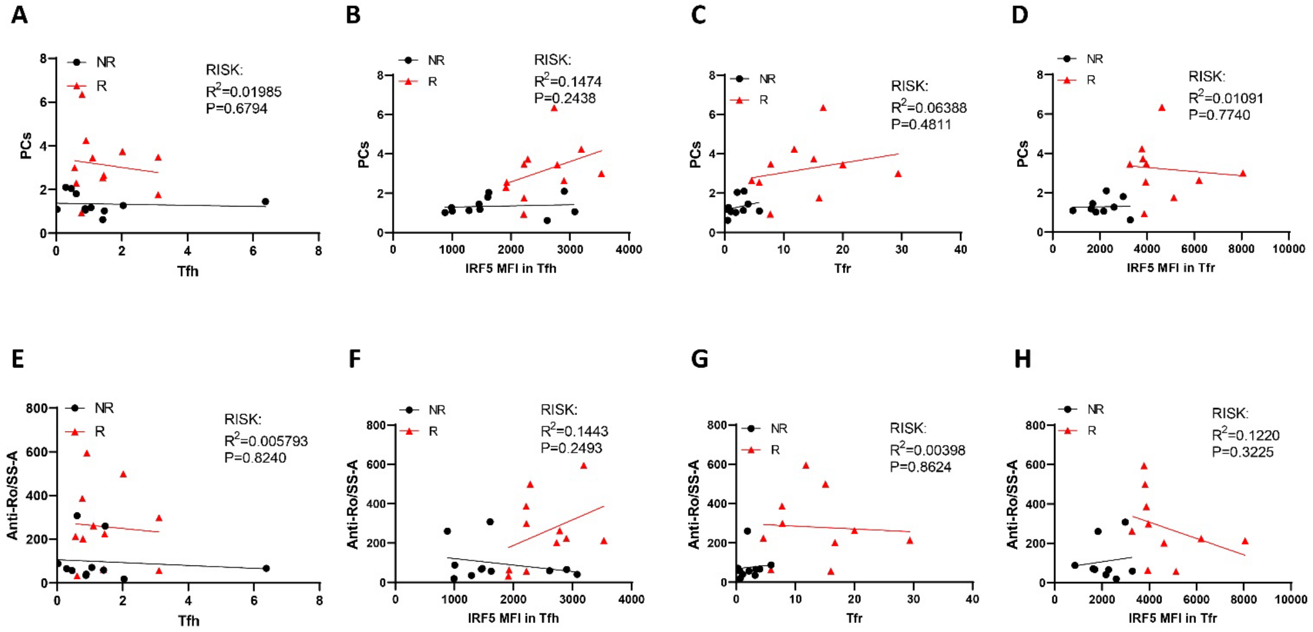

Supplemental Figure S2. Correlation analysis in healthy *IRF5*-SLE homozygous risk and non-risk carriers. **(A-D)** Correlations between the percentage of circulating PCs and CXCR5+PD1+Tfh **(A)**, IRF5 MFI in CXCR5+PD1+Tfh **(B)**, CXCR5+PD1+FoxP3+Tfr **(C)**, and IRF5 MFI in CXCR5+PD1+ FoxP3+Tfr **(D)**. **(E-H)** Correlations between Anti-Rho/SS-A autoantibodies and CXCR5+PD1+Tfh **(E)**, IRF5 MFI in CXCR5+PD1+Tfh **(F)**, CXCR5+PD1+FoxP3+Tfr **(G)**, and IRF5 MFI in CXCR5+PD1+ FoxP3+Tfr **(H)**. R^2 and P value determined using simple linear regression model.
